# Integrative biochemical and computational analysis identifies LOX-1 and IL-6R as therapeutic targets in oxidative, inflammatory and lipid dysregulation of ischemic stroke

**DOI:** 10.12669/pjms.42.8.15732

**Published:** 2026-08

**Authors:** Sabahat Fatima, Muhammad Sarwar, Madeeha Shahzad Lodhi, Sumaira Sharif

**Affiliations:** 1 Sabahat Fatima Department of Biochemistry, Gujranwala Medical College, Gujranwala, Pakistan, Institute of Molecular Biology and Biotechnology, The University of Lahore, Lahore, Pakistan; 2 Muhammad Sarwar, Institute of Molecular Biology and Biotechnology, The University of Lahore, Lahore, Pakistan; 3 Madeeha Shahzad Lodhi, Institute of Molecular Biology and Biotechnology, The University of Lahore, Lahore, Pakistan; 4 Sumaira Sharif, Institute of Molecular Biology and Biotechnology, The University of Lahore, Lahore, Pakistan

**Keywords:** Interleukin-6 receptor (IL-6R), Ischemic stroke, Lectin like oxidized LDL receptor-1 (LOX-1), Oxidized lipoproteins

## Abstract

**Background and Objective:**

Ischemic stroke (IS) being one of the leading causes of mortality and morbidity globally is initiated by complex interactions among lipid peroxidation, oxidative stress and metabolic dysfunction. This study aimed to identify therapeutic targets for these pathological pathways is critical for improving diagnostic and treatment strategies.

**Methodology:**

This case control study comprising 150 patients and one hundred healthy controls was conducted at DHQ Hospital, Gujranwala from June 2022 to June 2023. Biochemical analyses quantified apolipoproteins, oxidized lipoproteins (oxLDL, oxHDL), sdLDL, antioxidant factors (SOD), inflammatory (IL-6) and metabolic markers (ferritin, LDH) using ELISA and spectrophotometric assays. Moreover, molecular docking and 100 ns molecular dynamics (MD) simulations conducted to evaluate inhibitory potential and structural stability of selected small molecule ligands targeting major lipid related (LOX-1) and inflammatory (IL-6R) receptors.

**Results:**

The stroke patients demonstrated significantly elevated levels of biochemical markers and a marked reduction in SOD activity (p< 0.05). Multivariate regression modeling highlighted oxHDL, apoCIII, ferritin, and LDH as robust independent predictors of ischemic stroke. IL-6 displayed positive correlation with oxidized lipoproteins and apolipoproteins, supporting a link between inflammatory signaling and lipid abnormalities. Computational studies identified strong ligand receptor binding with docking energies ranging from −5.9 to −8.0 kcal/mol.

**Conclusion:**

Overall, integrated biochemical and computational approach highlights inflammatory activation, oxidative lipid damage, and iron dysregulation as vital contributors to stroke. Key receptors involved in lipid oxidation and cytokine signaling emerge as promising dual therapeutic targets, and the identified ligands show potential as lead candidates for future drug development against ischemic stroke.

## INTRODUCTION

Stroke is a major worldwide health burden affecting 107 per 100,000 individuals.^1^ The ischemic stroke (IS) or cerebral ischemia is the most prevalent (more than 80%) of all stroke types.^2^ Atherosclerosis considered as the main modulator with neuroinflammation, oxidative stress, and apoptosis as substantial contributors of stroke development and progression.^3^ Identification of reliable biochemical markers and molecular protein docking are crucial for early diagnosis and targeted therapeutic intervention for the disease. The lipoprotein abnormalities play a vital role with apolipoprotein CIII (apoCIII), apolipoprotein B (apoB) and atherogenic lipoproteins (oxLDL, sdLDL) driving the vascular damage.^4^-^7^ In addition, loss of antithrombotic and anti-inflammatory function of HDL leading to formation of dysfunctional or oxidized HDL (oxHDL) proposed as an evolving predictor of cardiovascular disease and ischemic stroke.^8^,^9^ However, the precise roles and interactions among these markers and cerebral infarction remained insufficiently characterized.

Lectin like oxLDL receptor-1 (LOX-1) has emerged as a newer biomarker and key regulator of atherosclerosis and stroke.^10^ Inflammation by promoting atherosclerosis found associated with stroke. IL-6, a key pro-inflammatory cytokine, is involved in atherosclerotic plaque progression and ischemic stroke.^11^ Binding of ligand with LOX-1 initiates release of proinflammatory cytokines, cell adhesion molecules and matrix metalloproteinases.^12^ However, there is limited data defining exact relationship of LOX-1 with interleukin-6 receptor (IL-6R) in ischemic stroke.

Oxidative stress and decreased activity of antioxidant enzyme, superoxide dismutase (SOD) play a critical role in the pathogenesis of ischemic stroke.^13^ An end product of purine metabolism, uric acid (UA), exhibits dual characteristics including pro-oxidant & an antioxidant function in cerebrovascular disease (CVD).^14^,^15^ Cerebral ischemia may be associated with high serum ferritin (Ferroptosis) through free radicals generated by this stored iron causing oxidative stress and hemorrhagic transformation risk.^16^,^17^ In addition to oxidative stress and inflammation, energy deficit (by vascular occlusion) reflected by altered lactate dehydrogenase (LDH)^18^ activity.

This study aimed to explore the complex interplay among oxidized lipoproteins, sdLDL, antioxidant markers, apolipoproteins (apoB, apoCIII), inflammatory predictors and ischemic stroke. Moreover, the in-silico protein-protein interaction analysis may direct to design interventions for prevention, early diagnosis and augment improved therapeutic targets for the disease.

## METHODOLOGY

The study included 150 ischemic stroke patients and one hundred healthy controls from Department of neurology, DHQ Hospital, Gujranwala from June 2022 to June 2023.

### Ethical Approval:

The Research Ethics Committee (Ref: IMBB/BBBC/22/536, Dated: March 14, 2022) of the Institute of Molecular Biology and Biotechnology (IMBB), University of Lahore (UOL) approved the current study.

All procedures were performed in accordance with the Declaration of Helsinki, and written informed consent obtained, before sample collection from all participants. Ischemic stroke diagnosis was confirmed using computed tomography (CT) or magnetic resonance imaging (MRI) scans. Patients with confirmed ischemic stroke with co-morbidities including hypertension, obesity and diabetes were involved, whereas individuals with cerebral hemorrhage, abscesses, head trauma, neoplasms, or severe cardiac and hepatic conditions were excluded. Demographic data, medical history, laboratory and clinical records were collected from all participants using a structured questionnaire. Physical examinations were conducted to determine systolic blood pressure (SBP), diastolic blood pressure (DBP) and body mass index (BMI). Venous blood samples collected from each participant, centrifuged for 15 minutes at 3400 revolutions per minute (rpm), and then stored at −80°C until further biochemical analysis. The biochemical parameters of conventional lipid profile including total cholesterol (TC), triglycerides (TG) and HDL concentrations were quantified using an enzymatic method.^19^

Friedewald equation by Dansethakul et al,^20^ used to calculate LDL and very low-density lipoprotein (VLDL) levels. The method defined by Srisawasdi^21^ used to estimate small dense LDL (sdLDL). Further, the levels of oxHDL, oxLDL, SOD, apoC-III, IL-6, ferritin and LDH assessed with the Human Oxidized High Density Lipoprotein ELISA Kit (Finetest, Cat. No. EH4858), Human Oxidized Low Density Lipoprotein ELISA Kit (Elabscience, Cat. No. E-EL-H6021), Human Superoxide Dismutase ELISA Kit (Elabscience, Cat. No. E-EL-H1113), Human Apolipoprotein CIII ELISA Kit (Elabscience, Cat. No. E-EL-H0467), Human IL-6 ELISA Kit (Elabscience, Cat. No. E-EL-H6156), Ferritin ELISA Test Kit (ANTEC Diagnostics, Ref. No. DS177720) and Diagnostic Kit for Lactate Dehydrogenase Activity (Bio-active Diagnostics, Ref. No. 104989993202) respectively. All ELISA assays tracked the sandwich principle, optical density (O.D) measured at 450 nm using a microplate reader. Uric acid concentration was measured using the spectrophotometric method^22^ while apolipoprotein B (apoB) levels were calculated using the equation described by Hwang et al.^23^ For statistical analysis, SPSS version 25, Minitab 22.2.1 and R 3.6.0+ was used.

The continuous variables stated as mean ± standard error of the mean (SEM). The statistical difference between controls and ischemic stroke patients for normally distributed variables assessed by Welch’s two sample t test and for multi group comparisons, analysis of variance (ANOVA) was used. The relationship among variables was examined by Pairwise Spearman’s correlation analysis. Multivariate logistic regression model used to designate independent predictors of ischemic stroke. A p-value < 0.05 considered as statistically significant. *In silico* workflow employed in the present study to investigate protein ligand interactions in ischemic stroke. Two vital target proteins selected after retrieving structures from Protein Data Bank.^24^

For this purpose, protein preparation completed by removal of ligands and water molecules, addition of missing side chains, addition of hydrogen atoms at physiological pH, kollman charge assignment in AutoDockTools 1.5.7^25^ and energy minimization using the CHARMM36,^26^ force field to permit structural stability. Further, selected compounds obtained from the PubChem^27^ and transformed into PDBQT for ligand preparation. Conformations of the ligands optimized and their energies were minimized using the MMFF94 force field. The potential active sites identified through literature validation using CASTp followed by grid box creation for precise docking. The lowest binding energy conformations with root mean square deviation (RMSD) <2 Å based validation were selected and Auto Dock Vina 1.2.3,^28^ used for performing molecular docking. Molecular dynamics (MD) simulations (100 ns, GROMACS^29^) evaluated stability under physiological conditions, incorporating ion neutralization, solvation and equilibration steps. Finally, trajectory analysis including RMSD, root mean square fluctuation (RMSF), radius of gyration (RoG) and hydrogen bonds confirmed the stability of protein ligand complexes with minor fluctuations primarily in flexible regions whereas core structures remained intact.

## RESULTS

The study comprised 250 participants including 150 ischemic stroke patients (79 males and 71 females) and 100 healthy controls (50 males and 50 females). There was not a significant difference of ages between the cases and the controls. The mean value of BMI was non significantly greater (p > 0.05) in ischemic stroke patients than in controls. There was a significant (p < 0.05) difference of systolic blood pressure (SBP) and diastolic blood pressure (DBP) between the two groups (Table-I). Biochemical analysis indicated significant differences between stroke patients and controls (Table-I).

**Table-I:** Baseline characteristics comparison between control and case group groups.

Parameters	Controls N = 100 Mean ± SEM	Cases N = 150 Mean ± SEM	p-value
Age(Yrs)	58± 1.1	58± 0.8	0.84
Body mass index (Kg/m^2^)	25± 0.3	28± 0.2	0.21
Systolic blood pressure (mm of Hg)	128± 1.5	144± 1.4	<0.001
Diastolic blood pressure (mm of Hg)	85± 1.3	94± 0.9	<0.001
Total Cholesterol (mg/dl)	176± 3.0	249± 5.6	<0.001
Triglyceride (mg/dl)	141± 4.8	223± 10.6	<0.001
High-density lipoprotein (mg/dl)	46± 0.8	34± 0.4	<0.001
Low-density lipoprotein (mg/dl)	110± 2.6	170± 5.2	<0.001
Very low-density lipoprotein (mg/dl)	27± 0.8	44± 2.1	<0.001
apolipoproteinB (mg/dl)	93± 2.0	144± 3.7	<0.001
apolipoproteinCIII (ng/ml)	521± 21.20	643± 26.8	0.003
small dense LDL (mg/dl)	36± 1.0	61±1.8	<0.001
oxHDL (ng/ml)	65± 3.3	153± 6.8	<0.001
oxLDL (ng/ml)	1077± 28.0	1270± 29.7	<0.001
Superoxide dismutase (pg/ml)	148± 3.4	63± 1.0	<0.001
Interleukin-6 (pg/ml)	3.0± 0.1	4.2± 0.1	0.008
Uric acid (mg/dl)	4.6± 0.1	5.8± 0.1	<0.001
Ferritin (ng/ml)	99± 5.1	151± 5.3	<0.001
Lactate dehydrogenase (U/L)	154± 5.3	247± 4.1	<0.001

Multivariate logistic regression analysis (Table-II) emphasized the biomarkers including oxHDL, oxLDL, sdLDL, apoB, apoCIII, IL-6, ferritin and LDH (p < 0.05 for all markers) as strong predictors of stroke, with oxHDL and apoCIII representing the highest associations. In addition, multiple linear regression modeling of IL-6 with atherogenic variables revealed strong associations with apoCIII, LDL, oxLDL, and sdLDL (p < 0.05 for all markers), while its association with uric acid was weak (p > 0.05). Interestingly, uric acid levels were significantly higher in patients (p < 0.05, Table-I), however, the regression analysis demonstrated a marginally non-significant p-value (p > 0.05), suggesting its limited role as a predictor of ischemic stroke.

**Table-II:** Results of Multivariate regression analysis of different variables with ischemic stroke.

Parameter	Coef	p-value	VIF
apoB	0.0401	0.001	1.01
apoC-III	0.00289	0.016	1.17
OxHDL	0.00168	0.000	1.16
OxLDL	0.00043	0.058	1.17
sdLDL	0.00687	0.000	1.32
Ferritin	0.01792	0.012	1.30
LDH	0.03741	0.000	1.35
IL-6	0.832	0.0008	1.55
Uric acid	0.398	0.075	1.10

Pairwise Spearman correlation exhibited a strong positive correlation between sdLDL and oxLDL (r = 0.837, p < 0.0001). In contrast, a weak correlation observed between sdLDL and oxHDL (r = 0.374, p < 0.0001). The correlation of SOD with oxidized lipoproteins was weak. Negligible associations between SOD and oxHDL (r = 0.183, p = 0.025), oxLDL (r = -0.258, p = 0.001), and sdLDL (r = -0.056, p = 0.429) were noted. A positive correlation between LDH and ferritin (r = 0.247, p = 0.002) suggested their involvement in disease pathology. Other associations, including those between apoB and ferritin or uric acid, were weak and statistically insignificant.

Based on mechanistic observations and correlations of biomarkers presented in the current study, two major proteins including Interleukin-6 Receptor (IL-6R) and Lectin-like oxidized LDL receptor-1 (LOX-1) were named as potential targets of non-covalent drug inhibition to alleviate the progression of ischemic stroke. IL-6R was selected as it proved central mediator of IL-6-mediated inflammation, highly increased in stroke patients and strongly linked with lipid abnormalities, including oxLDL, sdLDL, and apoCIII. Targeting IL-6R, may potentially inhibit inflammatory cascades that lead to vascular damage. The primary receptor, LOX-1, was selected as it promoted the internalization of oxidized lipids into vascular endothelium, leading to atherogenesis and plaque instability. Inhibition of LOX-1 may lead to decreased oxidative lipid deposition. Molecular docking of selected ligands with LOX-1 revealed variable binding affinities and interaction patterns providing insight into their inhibitory potential (Fig.2).

**Fig.1:**
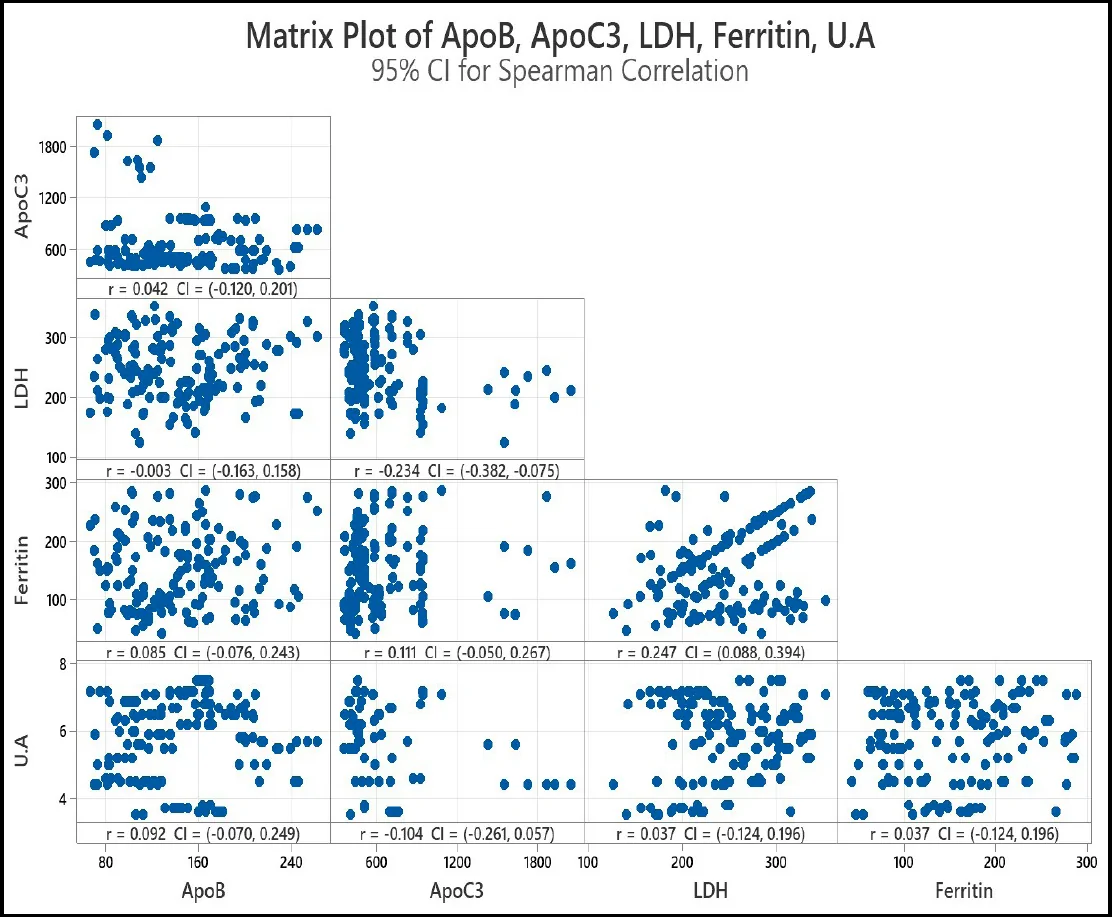
Relationship between apoB, apoC-III, Ferritin, LDH, Uric acid.

**Fig.2:**
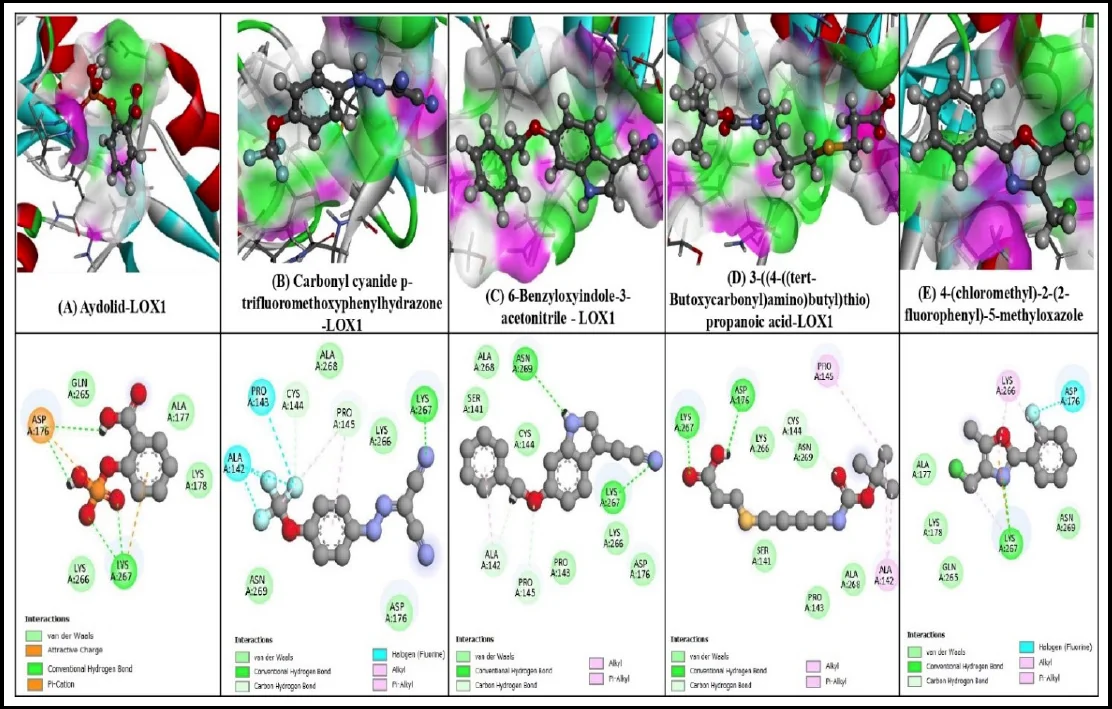
Molecular docking interactions of few selected ligands with LOX-1 (Lectin-like Oxidized LDL Receptor-1). (A) Aydolid, (B) 3-((4-((tert-Butoxycarbonyl)amino)butyl)thio)propanoic acid, (C) 6-Benzyloxyindole-3-acetonitrile, (D) Carbonyl cyanide p-trifluoromethoxyphenylhydrazone, and (E) 4-(Chloromethyl)-2-(2-fluorophenyl)-5-methyloxazole. The upper panels represent 3D binding conformations within the LOX-1 receptor active site, while the lower panels describe 2D interaction maps.

**Fig.3:**
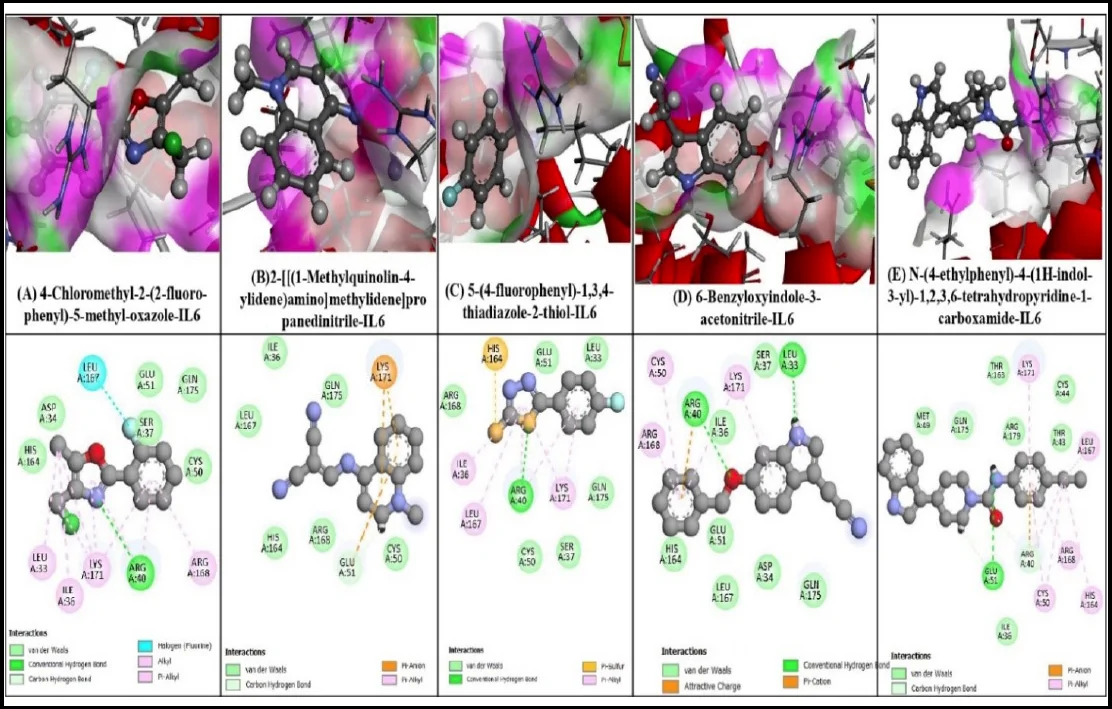
Molecular docking interactions of selected ligands with the IL-6 receptor. (A) 4-Chloromethyl-2-(2-fluorophenyl)-5-methyl-oxazole, (B) 6-Benzyloxyindole-3-acetonitrile, (C) N-(4-ethylphenyl)-4-(1H-indol-3-yl)-1,2,3,6-tetrahydropyridine-1-carboxamide, (D) 5-(4-fluorophenyl)-1,3,4-thiadiazole-2-thiol, and (E) 2-[[(1-Methylquinolin-4-ylidene)amino]methylidene]propanedinitrile. The top panels illustrate 3D binding conformations of each ligand within the IL-6 receptor pocket, while the lower panels depict 2D interaction maps.

Out of all the selected compounds, Aydolid, has a robust binding affinity of −7.33 kcal/mol and an RMSD of 3.46 Å through hydrogen bonding and electrostatic interactions. The weakest binding affinity was detected with the fluorophenyl-methyloxazole derivative (−5.96 kcal/mol), though its low RMSD of 1.70 Å specified a highly stable docking pose. The docking analysis of few selected ligands with the IL-6 receptor confirmed strong binding affinities, with various compounds exhibiting stable interactions within the active site (Fig.3). The 4-chloromethyl-2-(2-fluorophenyl)-5-methyl-oxazole revealed the highest binding affinity of −8.05 kcal/mol with a low RMSD (1.91 Å), indicating a stable docking pose reinforced by conventional hydrogen bonds. Finally, the quinoline-based derivative bound with the weakest affinity (−7.27 kcal/mol, RMSD 2.39 Å), stabilized through hydrogen bonds as well as π–cation and π–alkyl interactions alongside van der walls contacts.

In MD simulation, the RMSD plot, provided a detailed overview of the backbone stability of LOX upon complexation with each of the ten ligands that displayed a rapid equilibration phase within the first 5–15 nanoseconds (ns). The average RMSD for most systems ranges between 1.5 and 2.3 Å, with overall variations confined within 1.0–3.0 Å, indicating good conformational stability. The absence of continuous upward drift in all trajectories confirmed that LOX remained stable and that none of the ligands induced global unfolding or loss of structural integrity.

**Fig.4:**
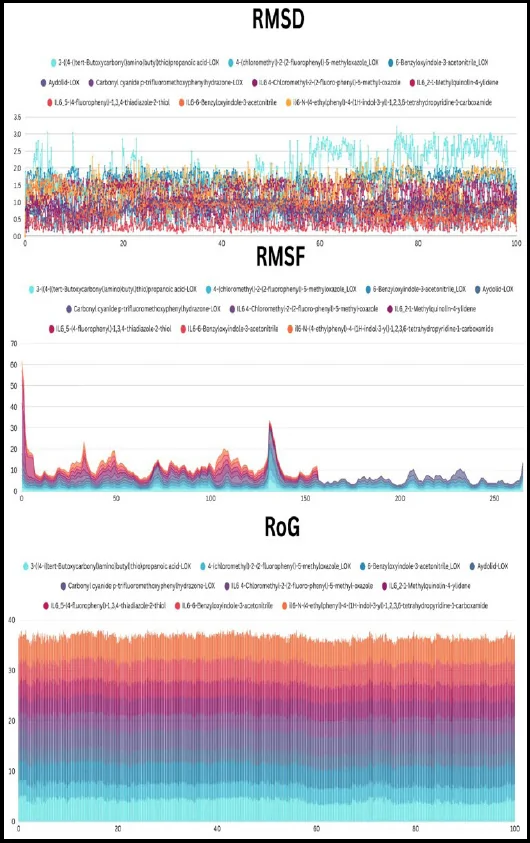
Structural stability analysis of LOX–ligand complexes over 100 ns molecular dynamics simulations. (A) RMSD profiles indicate that all LOX–ligand systems equilibrate rapidly and remain stable throughout the simulation. (B) RMSF plots reveal that flexibility is confined to terminal and loop regions. (C) The radius of gyration (RoG) remains constant around 34–36 Å for all complexes.

The Root Mean Square Fluctuation (RMSF) analysis (Fig.4) depicted that LOX–ligand complexes maintain overall structural rigidity, with lowest fluctuations (<5 Å) in core and catalytic regions, indicating stable secondary structures. Higher flexibility observed in loop and terminal regions (residues 15– 35, 55– 85, 120– 145 and 200– 225), especially around residues 120–145, representing normal surface mobility. Interestingly, molecular ligands including IL6-N-(4-ethylphenyl)-4′-(1H-indol-3-yl)-1,2,3,6-tetrahydropyridine-1′-carboxamide and IL6-4-chloromethyl-2-(2-fluorophenyl)-5-methyloxazole revealed reduced flexibility pointing enhanced stabilization. Importantly, no extreme fluctuations (>7 Å) noted, confirming absence of structural instability.

The Radius of Gyration (RoG) plot (Fig.4) highlighted maintenance of compact LOX- ligand complex stability throughout 100 ns simulation period. All systems remarkably exhibit stable radius of gyration profiles that remained nearly constant throughout the trajectory. The RoG values centered around 34–36 Å, with minimal fluctuations (±0.5 Å). The little oscillations observed are likely due to natural breathing signals of the protein and do not indicate structural instability. Overall, the RoG study augmented the RMSD and RMSF findings, confirming that LOX maintains a firmly packed, native like conformation upon binding all tested ligands.

## DISCUSSION

Ischemic stroke being a major cause of morbidity and mortality worldwide exert a huge economic burden on the state, especially in developing countries including Pakistan. More recent studies have focused on recognizing biomarkers that can empower early diagnosis and improve treatment strategies for the disease. This study systematically assessed the relationship between different biomarkers and ischemic stroke. For instance, present study highlighted significantly elevated levels of apoB and apoCIII in ischemic stroke patients compared to controls (p = 0.000), in accordance with previous work demonstrating the role of apoCIII and apoB in increasing the likelihood of cerebrovascular events.^4^,^30^ However, these are in comparison with the study indicating lower apoCIII levels in patients with macrovascular disease.^31^

In addition, oxidized lipoproteins (oxLDL, oxHDL), sdLDL found significantly higher in ischemic stroke patients. Although the role of oxLDL and sdLDL in atherosclerosis and cerebrovascular disease highlighted in earlier studies.^6^,^7^ However, the current study is one of the first to report a direct association between oxHDL and ischemic stroke suggesting that oxHDL may serve as an early screening biomarker for ischemic stroke. A strong positive correlation noted between oxLDL and sdLDL (r = 0.837, p = 0.000), consistent with previous work linking raised oxLDL with elevated sdLDL levels.^32^

The current research project demonstrated significantly lower SOD level in patients of stroke consistent with previous studies that linked decreased SOD to poor post stroke outcomes.^13^ However, a weak correlation between SOD levels and oxidized lipoproteins in this study suggests that additional oxidative pathways may be involved in lipoprotein oxidation and vascular occlusion. Limited data is available regarding a positive correlation between oxLDL and SOD in hypercholesterolemic patients.^33^ Uric acid, that plays dual role as a pro-oxidant as well as an antioxidant, found significantly higher in stroke patients.

However, regression analysis did not label it as a strong predictor of stroke risk. This is in alignment with previous work that has presented conflicting evidence regarding the pro- oxidant or antioxidant role of uric acid in cerebrovascular disease.^14^,^15^,^34^ Ferritin, an iron storage protein, found significantly higher in ischemic stroke patients (p = 0.012) as compared to controls. These findings are consistent with recent studies demonstrating a strong positive correlation between ferritin levels and both stroke severity and prognosis.^16^,^17^

However, a positive correlation between ferritin and LDH levels observed in the present study has not reported previously. This relationship indicated contribution of iron overload to metabolic stress and neuronal injury in ischemic stroke. LDH, an important enzyme in anaerobic metabolism, found significantly increased in ischemic stroke patients (p = 0.000), consistent with previous research linking increased LDH levels to a higher risk of ischemic stroke and worse clinical outcomes.^19^,^35^ However, a recent study reported an inverse relationship between LDH and ischemic stroke, possibly owing to differences in methodology and sample size.^36^ IL-6, being an inflammation promoting cytokine, was significantly raised in patients of ischemic stroke and strong associations of IL-6 with LDL, oxLDL, sdLDL and apoCIII were noted.

These results support previous findings that link increased IL-6 to progression of atherosclerosis and poor stroke outcomes, reinforcing its potential as a therapeutic target in stroke management.^11^,^37^ Moreover, molecular docking and simulation studies indicated that LOX maintained its structural and global stability with all ten ligands. The fluctuations of RMSD remained in the range of 1–3 Å, the RMSF changed only with respect to the flexible loops and the ends, leaving the catalytic core intact, and the RoG remained around 35 Å with only small changes. This study indicated that all ligand–LOX complexes were stable in motion, compact in structure, and in reasonably consistent form. This is interpreted as the ligand systems across different complexes introduce no incompatibility with LOX’s native fold, and may even provide some degree of stabilization. Such stable molecules further enable the pursuit of equilibrium for analyzing hydrogen bond occupancy, binding free energy changes and bonded structures, as well as dynamic interaction mapping for making the analysis reliable.

The biomarkers (oxHDL, apoCIII, sdLDL, ferritin, IL-6 and LDH) and their novel correlations identified in the current study may carry extensive clinical implication in risk stratification, early diagnostic analysis and therapeutic intervention. The inclusion of these markers in conventional laboratory testing panels after thorough multi centered, in- vitro studies, may enhance sensitivity and specificity of stroke risk assessment. Moreover, the results of simulation studies indicating various ligands as molecular targets against LOX-1 and IL-6R could direct towards therapeutic options after widespread animal model studies and clinical trials.

### Strengths of the study:

This report is the first of its kind integrating biochemical and bioinformatic tools to explore pathogenesis and targeted therapy of ischemic stroke in patients from this region.

### Limitations:

The study was limited by time constraints (modest sample size), single centered basis and case control design.

## CONCLUSION

This work highlighted elevated oxidized lipoprotein (oxLDL, oxHDL), sdLDL, apolipoprotein (apoB, apoCIII), IL-6, ferritin, LDH, and uric acid levels, coupled with declined SOD activity manifesting the disruption of lipid metabolism, the antioxidant complex, and the inflammatory axis. The bioinformatics and molecular simulation study strengthened the clinical work by showing that IL-6R and LOX-1 as key molecular targets with selected ligands (fluorophenyl-oxazole derivatives and Aydolid) depicting strong binding affinity and structural stability. Collectively, integrated biochemical and computational techniques provided the basis for novel diagnostic and therapeutic approaches in ischemic stroke.

### Authors’ Contribution:

**SF:** Project design, acquisition of data, writing of manuscript, analysis and interpretation.

**MS:** Revision and critical analysis of the manuscript.

**MSL:** Critical review**,** Analysis and discussion.

**SS:** Design of study, critical analysis of data, revising the manuscript.

The corresponding Author is responsible for the accuracy and integrity of work.
